# Comparison of joint position sense measured by inertial sensors embedded in portable digital devices with different masses

**DOI:** 10.3389/fnins.2025.1561241

**Published:** 2025-05-13

**Authors:** José Ramon Gama Almeida, Luis Carlos Pereira Monteiro, Pedro Henrique Castro de Souza, André dos Santos Cabral, Anderson Belgamo, Anselmo de Athayde Costa e Silva, Alex Crisp, Bianca Callegari, Aymee Lobato Brito, Paulo Eduardo Santos Ávila, José Aparecido da Silva, Gilmara de Nazareth Bastos, Givago Silva Souza

**Affiliations:** ^1^Instituto de Ciências Biológicas, Universidade Federal do Pará, Belém, Brazil; ^2^Instituto de Tecnologia, Universidade Federal do Pará, Belém, Brazil; ^3^Centro de Ciências Biológicas e da Saúde, Universidade do Estado do Pará, Belém, Brazil; ^4^Instituto Federal de São Paulo, Piracicaba, Brazil; ^5^Instituto de Ciências da Educação, Universidade Federal do Pará, Belém, Brazil; ^6^Instituto de Ciências da Saúde, Universidade Federal do Pará, Belém, Brazil; ^7^Postgraduate Program in Behavioral Sciences, University of Brasília, Brasília, Brazil; ^8^Núcleo de Medicina Tropical, Universidade Federal do Pará, Belém, Brazil

**Keywords:** proprioception, joint sense position, smartphone, wearable sensor, digital health, mHealth

## Abstract

**Background:**

Joint position sense can be assessed using various devices, including inertial sensors embedded in smartphones and wearable technologies. However, the mass of these portable instruments may influence proprioceptive input during joint repositioning tasks.

**Purpose:**

This study aimed to compare participants’ performance in a joint position sense task using a smartphone and an ultra-light wearable sensor to measure elbow angular displacement.

**Methods:**

Sixteen adults participated in a passive-active joint position sense test. In this task, participants were required to memorize a passively flexed elbow position and actively reposition the joint across four trials. The angular position during joint repositioning, as well as absolute and relative errors, were compared between trials using a smartphone (weighing several hundred grams) and an ultra-light wearable sensor (weighing only a few dozen grams). Agreement analysis between the devices and reliability assessments for inter-device measurements and for each device were conducted.

**Results:**

No significant variation in the joint angle at the target position was observed across trials using the ultra-light wearable sensor. In contrast, a significant increase in joint angle at the target position was noted when the smartphone was used. Absolute errors were similar between devices, while relative errors showed significant differences in the first two trials. Overall, systematic biases favored the measurements obtained with the smartphone and inter-device reliability were moderate. Smartphone demonstrated moderate-to-good reliability, and the wearable had poor-to-moderate in test–retest evaluation.

**Conclusion:**

Although measurements from the two devices showed agreement, significant systematic biases were observed, favoring the heavier device. Both the smartphone and the wearable sensor provided reliable measurements for assessing elbow joint position sense.

## Introduction

As described by Sherrington (1935), proprioception refers to the ability to perceive joint movement and positioning in space, even in the absence of visual feedback (Ager et al., 2017). This ability is based on sensory signals from muscle, joint, and skin receptors, which are processed by the brain to determine body position and movement (Goble, 2010; Proske, 2023). Initially focused on the basic perception of position and movement, over time the definition of proprioception has been expanded to encompass concepts such as kinesthesia, the perception of active and passive joint movement, joint position sense, the active or passive reproduction of joint angles, and the ability to detect vibrations (Ager et al., 2017; Smith et al., 2020).

The assessment of joint position sense (JPS) varies significantly depending on whether the reference joint positioning is performed actively or passively, due to the different contributions of muscle spindles and joint receptors (Proske, 2023; Proske and Weber, 2023). In active reference positioning, the participant uses voluntary contractions to position the joint, involving central motor commands and muscular effort (Chrysagis et al., 2021). This approach may lead to a position perception influenced by the interpretation of the effort required to achieve or maintain the position (Roach et al., 2023). Conversely, in passive reference positioning, the joint is moved or held in place by external forces without voluntary muscle activation (Chrysagis et al., 2021; Han et al., 2016). Under this condition, position perception predominantly relies on signaling from muscle spindles and, at the limits of joint movement, from joint receptors (Riemann and Lephart, 2002). Roach et al. (2023) demonstrated that during active elbow flexion repositioning following a passive reference positioning, repositioning errors were close to zero degrees. As a result, joint repositioning did not show significant deviations toward more flexed or extended positions when compared to the passive reference positioning (Jebreen et al., 2023).

Currently, several tools are used to assess proprioception, including isokinetic devices, evaluation platforms, wearable inertial sensors, and sensors integrated into smartphones (Tsay et al., 2014; Chiyohara et al., 2023; Chirumbole et al., 2024; Horváth et al., 2024). Among these, the use of portable inertial sensors stands out as an innovative and practical approach capable of providing precise measurements of joint angular displacements (Onoda and Huo, 2016). Moreover, these tools have the potential to enhance the accessibility of proprioceptive assessments to a broader audience due to their low financial cost.

However, when considering the use of portable digital devices to measure JPS, the question arises regarding the comparability between smartphone-attached sensors and ultra-light wearable sensors (Costa et al., 2020). Both types of devices require attachment to the body to measure angular displacements, which involves differing weights being affixed to the movable structure of the joint positioning (Beshara et al., 2020). This factor may influence the activation of articular, muscular, and cutaneous proprioceptive receptors, potentially affecting the perception of joint positioning. Therefore, the impact of device weight and type on measurement accuracy should be considered (Goble et al., 2012). Existing evidence of the influence of additional load during the repositioning task on proprioceptive acuity has been reported (Suprak et al., 2007). Muscle contraction associated with the increasing weight of the object is one of the central factors influencing the ability to discriminate weights and proprioceptive acuity in general, and the sensitivity of myotendinous mechanoreceptors is increased in situations of greater muscle activation because when tension increases, the stimulation of Ib afferent fibers increases concomitantly (Gregory et al., 2002).

Our group has shown that certain sensorimotor functions, such as hand tremor and anticipatory postural adjustments, when assessed using inertial sensors embedded in wearable devices and smartphones, may exhibit differences in their characterization, likely due to the influence of the sensor weight (Moraes et al., 2023; Santos et al., 2022). Since smartphones have greater mass compared to ultra-light wearable sensors, their use for recording joint repositioning may amplify differences in proprioceptive activation between active and passive positioning conditions.

The present study aimed to compare active elbow flexion repositioning values obtained using ultra-light wearable and smartphone, as well as the agreement between their measurements and the reliability of both devices to evaluate the JPS.

## Materials and methods

### Ethical considerations

This study was approved by the Human Research Ethics Committee of the Tropical Medicine Center of the Federal University of Pará (#6.871.513). All participants signed the informed consent form after the experimental procedures were explained, agreeing whether or not to participate in the research to make up the sample.

### Participants

The present study employed a quantitative, analytical, and cross-sectional design, utilizing a convenience sample of 16 healthy adults (10 women and 6 men; age: 26.0 ± 6.29 years; height: 1.67 ± 0.09 m; weight: 70.24 ± 14.38 kg; body mass index: 25.0 ± 4.46 kg/m^2^). None of the participants had a history of chronic, degenerative diseases or conditions that could impair motor performance during the experiments.

The inclusion criteria comprised healthy adults aged between 18 and 50 years without chronic-degenerative, neurological, or orthopedic conditions affecting the upper limbs. Individuals presenting any musculoskeletal symptoms, such as pain, injuries, surgeries, injections, or paresthesia in the upper limbs within the 6 months prior to the study, were excluded.

### Experimental protocol

The technique selected to assess proprioceptive performance was the joint position sense (Chrysagis et al., 2021; Goble, 2010; Han et al., 2016), using the ipsilateral passive-active repositioning method (Gillespie et al., 2016). To measure joint range of motion, a 173 g smartphone (Xiaomi Redmi Mi9, Beijing) and a 6 g ultra-light wearable inertial sensor (Meta Motion C Sensor, mBientLab, San Francisco, USA) were employed. We created a velcro sleev (~50 g) to hold the smartphone in the distal arm, while we used a rubber wristband of 11 g was used to fixate the ultra-light wearable inertial sensor in the limb. The smartphone set had around 220 g and the ultra-light wearable set had about 17 g.

An Android-based application (Momentum Sensors, Belém, Brazil) was used to record accelerometer readings from the smartphone at a sampling rate of 50 Hz. Simultaneously, the MetaBase application (mBientLab) controlled the acquisition and storage of accelerometric data from the inertial sensor at a sampling rate of 100 Hz. Both the smartphone and the inertial sensor were affixed to the dorsal region of the distal third of the participants’ forearm (Ramos et al., 2019).

The testing procedure was standardized for recordings performed with both devices. Initially, the experimenter passively positioned the participant’s elbow at 90° of flexion with the assistance of a handheld goniometer, maintaining this position for 10 s. Subsequently, the joint was returned to full extension. Following the initial positioning, the participant, with eyes closed, was verbally instructed to actively reposition the elbow to the previously demonstrated flexion position and maintain it for 10 s. Subsequently, the participant actively repositioned the elbow to full extension, also maintaining it for 10 s.

**Figure 1:**
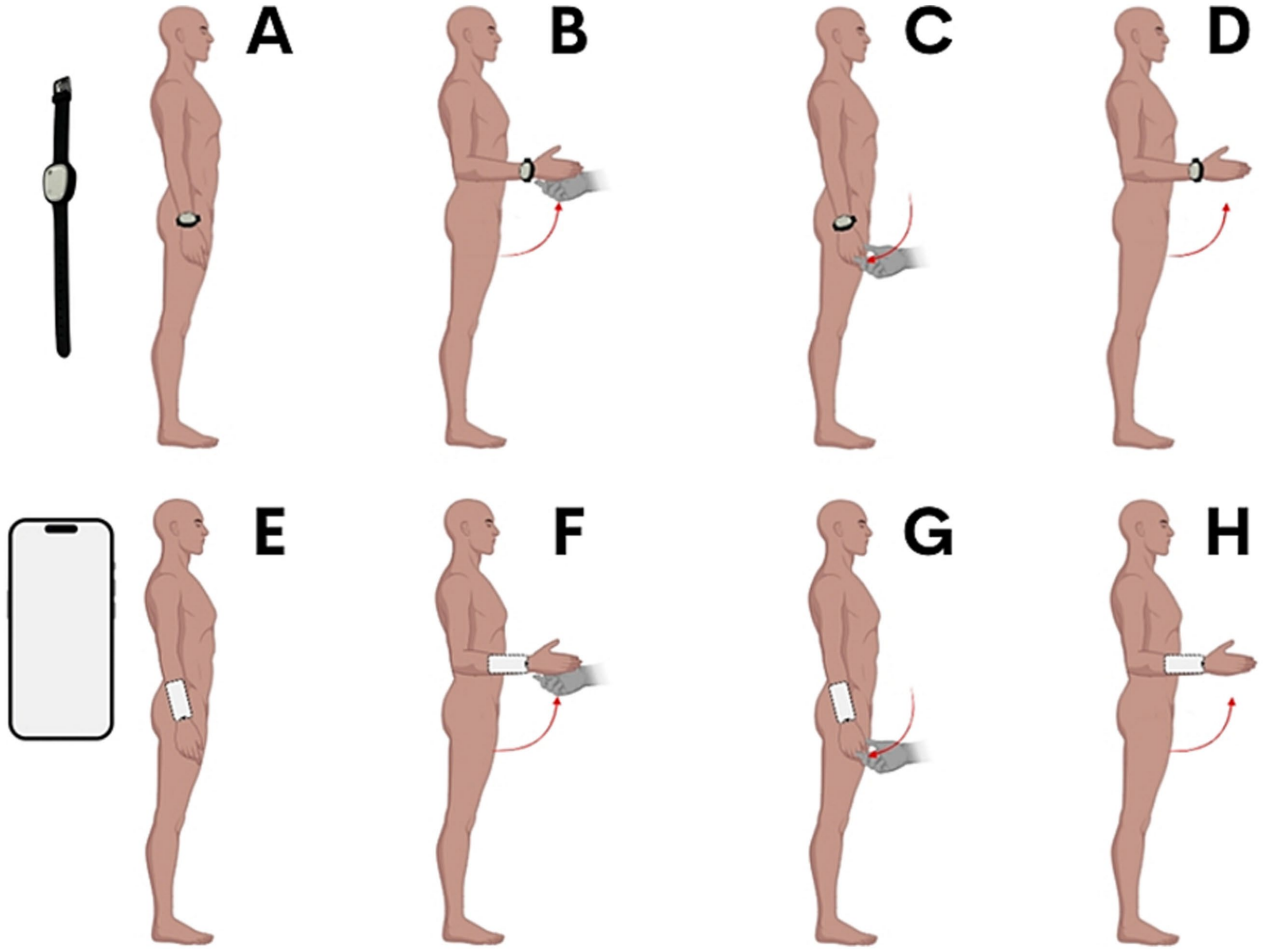
Experimental procedures. For both instruments, the same procedures were applied. Steps **(A–D)** describe the process using the ultra-light wearable sensor, while steps **(E–H)** detail the procedure with the smartphone. The test began with the participant’s elbow fully extended, which was then passively moved by the experimenter to a flexed position of 90°. This position was held for 10 s, during which the participant was instructed to memorize it. Subsequently, the participant actively repositioned their elbow to the reference position across four trials.

This repositioning cycle was repeated four consecutive times, first with the smartphone and then with the sensor, with a rest period between the tests with each device. Figure 1 shows a schematic representation of the procedures for both devices.

Each participant underwent a familiarization session prior to data collection (Chrysagis et al., 2021). The same protocol and procedure were applied using both the smartphone and the wearable inertial sensor, with a rest interval of approximately 1 min between tests.

### Data analysis

Both recording applications exported the accelerometric time series as “.csv” files for offline signal processing, which was performed using routines developed in Python programming language. The accelerometric signals were processed with a 40 Hz low-pass filter (4th-order Butterworth filter) with zero phase delay and interpolated to a frequency of 100 Hz. Joint angles (in degrees) were calculated according to Equation 1 (Pedley, 2013).


(1) $$\text{Angle}=90+\arctan(y/\sqrt{x^{2}+z^{2})}*180/\pi$$

where *x*, *y*, and *z* represent the time series of the smartphone’s *x*, *y*, and *z* axes, *π* is the mathematical constant pi, and arctan is the inverse tangent calculated.

As a calibration step for the angular measurements using both devices, we used manual goniometric assessment of the participant’s full elbow extension to calculate angular variations from this reference position. In Supplementary material 1, we present a comparison of the angular values obtained using the smartphone and a 3D motion capture system (unpublished data). This step was taken to verify whether the smartphone’s positioning (or wearable device) might compromise the accuracy of joint movement angle calculations, given that the axes of the accelerometer may not always perfectly align with the anatomical axes of the forearm. The waveforms recorded by both instruments showed similar patterns. When comparing the mean angular measurements from each device (smartphone: 90.63 ± 9.64 degrees; video capture system: 85.46 ± 8.86 degrees), a significant difference was observed (t (79) = 6.51, *p* < 0.0001, 95% confidence interval = [2.64, 4.92]); however, the smartphone’s measurements were closer to the target joint angle.

Most of the signal’s energy obtained from both devices concentrated at frequencies below 1 Hz, while frequencies above this threshold contribute minimally to the overall signal energy. Nevertheless, a 40-Hz cutoff frequency was chosen for the following reasons: (i) the present study is among the first to explore the use of mobile devices for assessing joint position sense. Therefore, we opted to preserve most of the signal’s spectral content until a consensus on appropriate filtering characteristics for this type of study emerges. Supplementary material 1 provides a figure illustrating the signal’s energy spectrum and another comparing the signals filtered at 40 Hz (as used in this study), 10 Hz, and 1 Hz, demonstrating the minimal differences between these filtering conditions; (ii) Since the event analyzed in the present study (active joint repositioning) approximates a square wave, characterized by a spectrum dominated by odd harmonics, applying excessively low cutoff frequencies can distort the waveform, as also illustrated in Supplementary Figure S2; (iii) Additionally, it is worth noting that a zero-phase filter was employed to enhance the methodological rigor of data analysis, despite the absence of transient, stimulus-synchronized components in the current study. In contexts where precise timing is critical for joint angle estimation and error calculation, this filtering approach becomes even more relevant.

The average joint angle was calculated for the baseline and for each trial within the interval from 1 to 9 s of the elbow flexion period. The relative error and absolute error of active repositioning were calculated. The relative error was considered the difference between the angular values of active repositioning and the passive reference positioning, while the absolute error was the absolute value of this same difference.

### Statistical analysis

All statistical analyses were conducted using R language. The stats, car, and ez packages were used for statistical analyses. The data distribution was assessed for normality using the Shapiro–Wilk test. A paired Student’s t-test was also used to compare the angular values, relative errors, and absolute errors measured with both instruments at each different phase of the experiment. Additionally, a one-way repeated measures ANOVA was performed to analyze the data. Subsequently, the angular values of the elbow in the baseline condition, as well as the angular values, relative errors, and absolute errors of the four trials for recordings made with each device, were compared using one-way repeated measures ANOVA. The assumptions of the ANOVA test were verified as follows: the normality of residuals was assessed using the Shapiro–Wilk test and visual inspection of Q–Q plots; homogeneity of variances was evaluated using Bartlett’s test; and sphericity was assessed using Mauchly’s test. In the significant results, we calculated the effect size (Cohen’s d for paired t-test, and Cohen’s f for one-way repeated measures ANOVA) and the achieved power.

We conducted an agreement analysis consisting of a Bland–Altman analysis and the calculation of the intraclass correlation coefficient (ICC) between the measurements obtained from both devices. These analyses were performed for joint angle, absolute error, and relative error across different conditions (baseline, trial #1, trial #2, trial #3, and trial #4).

In the Bland–Altman analysis, we computed the mean bias (i.e., the average difference between devices), the limits of agreement (defined as the bias ± 1.96 × the standard deviation of the differences), and the agreement range (calculated as the difference between the upper and lower limits of agreement). A bias was considered statistically significant when zero fell outside the 95% confidence interval of the mean bias.

ICC estimates and their 95% confidence intervals were calculated using R software (psych package), based on single measurements, absolute agreement, and a two-way random-effects model (ICC [2,1]). Additionally, we estimated the ICC and their 95% confidence intervals using a two-way mixed-effects model based on single measurement and absolute agreement (ICC [3,1]) to assess the reliability of each device. Standard error of measurement (SEM) and Minimal Detectable Change (MDC) were also calculated to quantify the precision and meaningfulness of the measurement of each device.

ICC results were classified as follows: ICC < 0.5 = poor reliability; 0.5–0.75 = moderate reliability; 0.75–0.9 = good reliability; and ICC > 0.9 = excellent reliability (Koo and Li, 2016; Portney and Watkins, 2000).

A significance level of 5% was adopted for all statistical procedures.

## Results

**Figure 2:**
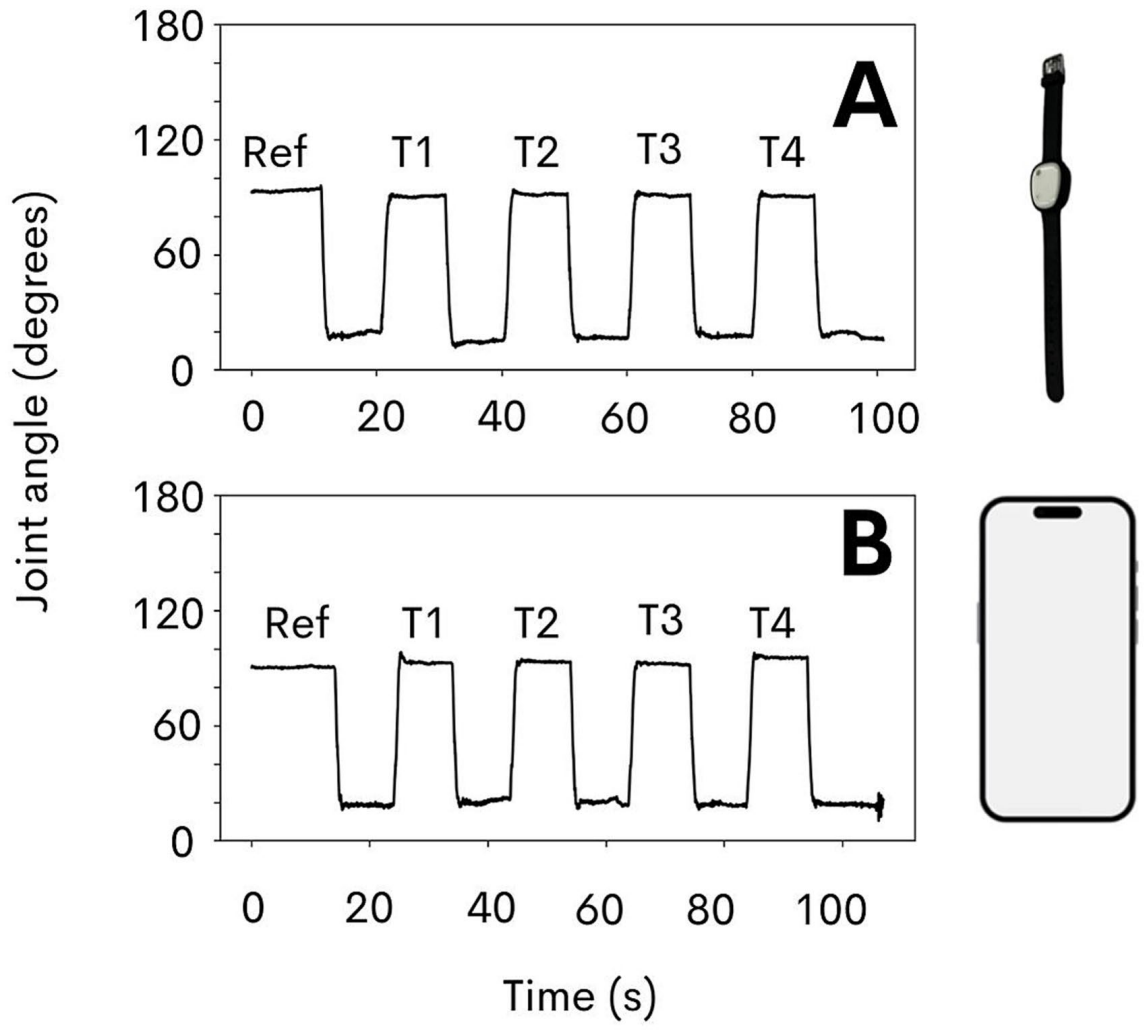
Inertial recordings of the angular position of the elbow during the experiments carried out using the ultra-light wearable sensor **(A)** and smartphone **(B)** of a same representative participant of the sample. Ref: reference position; T1, T2, T3, and T4 represent each active trial.

Figure 2 presents the recordings obtained with both device (smartphone and ultra-light wearable sensor) for a representative participant from the sample.

Table 1 presents the angular values, absolute error, and relative error measured at baseline and during the active joint repositioning trials with both instruments. The angular values in the baseline condition, obtained during the tests with the ultra-light wearable sensor and the smartphone, showed no statistically significant difference (*p* > 0.05), indicating that the passive positioning performed by the experimenter was consistent across both instruments.

**Table 1:** Comparison of baseline and repositioning attempts in the JPS test using smartphone and ultralight wearable sensor.

	Baseline	T1	T2	T3	T4	*p*-value (ANOVA)
Valor angular (deg)						
Wearable sensor	94.5 ± 1.88	94.2 ± 3.35	94.3 ± 3.27	95.1 ± 4.09	95.5 ± 5.11	>0.05
Smartphone	94.3 ± 2.55	96.4 ± 4.04	97.2 ± 4.83	97.7 ± 5.10	97.7 ± 5.78	<0.01*
*p*-value (t-test)	>0.05	0.11	0.056	0.13	0.26	–
Absolute error (deg)						
Wearable sensor	–	1.93 ± 1.98	2.30 ± 1.65	2.51 ± 2.06	3.63 ± 2.35	>0.05
Smartphone	–	2.75 ± 2.95	3.32 ± 3.84	3.96 ± 3.95	4.16 ± 3.75	>0.05
*p*-value (t-test)	–	0.37	0.34	0.2	0.64	–
Relative error (deg)						
Wearable sensor	–	−0.29 ± 2.8	0.14 ± 2.89	0.65 ± 3.25	0.98 ± 4.31	0.1
Smartphone	–	2.10 ± 3.47	2.98 ± 4.13	3.44 ± 4.43	3.42 ± 4.48	0.1
*p*-value (t-test)	–	0.04*	0.02*	0.05	0.12	–

T1, first trial; T2, second trial; T3, third trial; T4, fourth trial. *: significant difference (*p* < 0.05).

Similarly, the angular values estimated in each of the four trials showed no significant differences between the measurements made with the ultra-light wearable sensor and the smartphone. However, when comparing the angular values between the different trials performed with the same device, it was observed that with the ultra-light wearable sensor, there were no significant differences between the baseline and subsequent trials, nor between the individual trials themselves. On the other hand, when the smartphone was used, a significant increase in angular values was observed during joint repositioning (*p* < 0.01, Cohen’s *f* = 1.6, achieved power = 99%).

The comparison of absolute errors between the same test conditions performed with both devices did not reveal significant differences. Similarly, no significant differences were identified in the absolute errors estimated between the trials conducted with the same device. On the other hand, the comparison of relative errors estimated with both devices in the first two trials showed significant differences, with tests using the smartphone exhibiting larger errors than those performed with the ultra-light wearable sensor (t1: *p* < 0.05, Cohen’s d = 0.75, achieved power = 80%; t2: *p* < 0.05, Cohen’s d = 0.88, achieved power = 90%). The comparison of relative errors in trials 3 and 4 indicated similar values between the two devices (*p* > 0.05). Additionally, no significant differences were observed in the absolute errors estimated between the trials performed with the same device (*p* > 0.05).

### Agreement between measurements

**Figure 3:**
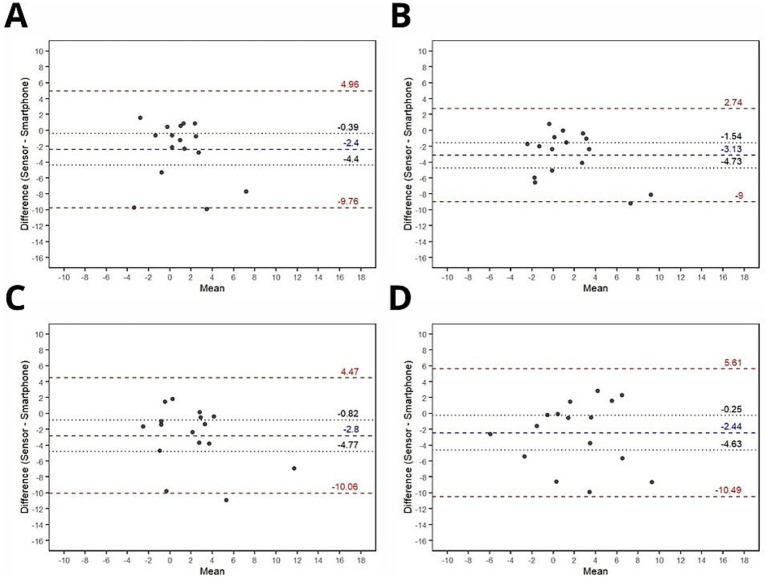
Bland-Altman plots for agreement analysis of absolute error between smartphone and ultra-light wearable sensor measurements. **(A)** Absolute error (Trial 1); **(B)** Absolute error (Trial 2); **(C)** Absolute error (Trial 3); **(D)** Absolute error (Trial 4) Blue dashed lines: Mean bias (systematic difference between devices) Red dashed lines: 95% limits of agreement (bias ± 1.96 × SD of differences). Black dotted lines: 95% confidence intervals for bias and limits.

**Figure 4:**
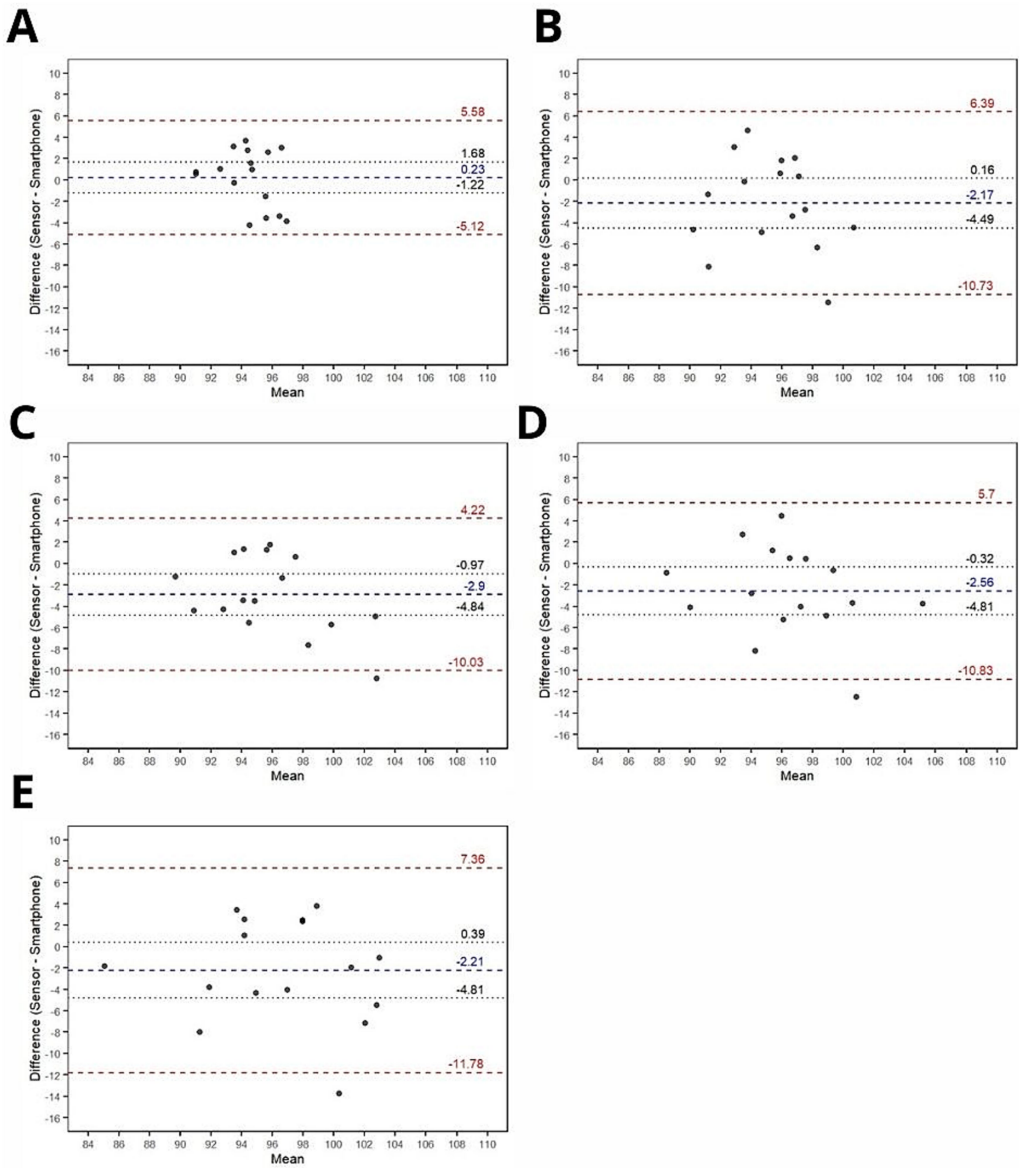
Bland–Altman plots for agreement analysis of absolute joint angle errors (°) between smartphone and ultra-light wearable sensor measurements. (A) Baseline; (B) Trial 1; (C) Trial 2; (D) Trial 3; (E) Trial 4. Blue dashed lines: Bias (mean difference between devices). Red dashed lines: Limits of agreement (bias ± 1.96 × SD). Black dotted lines: 95% confidence intervals.

**Figure 5:**
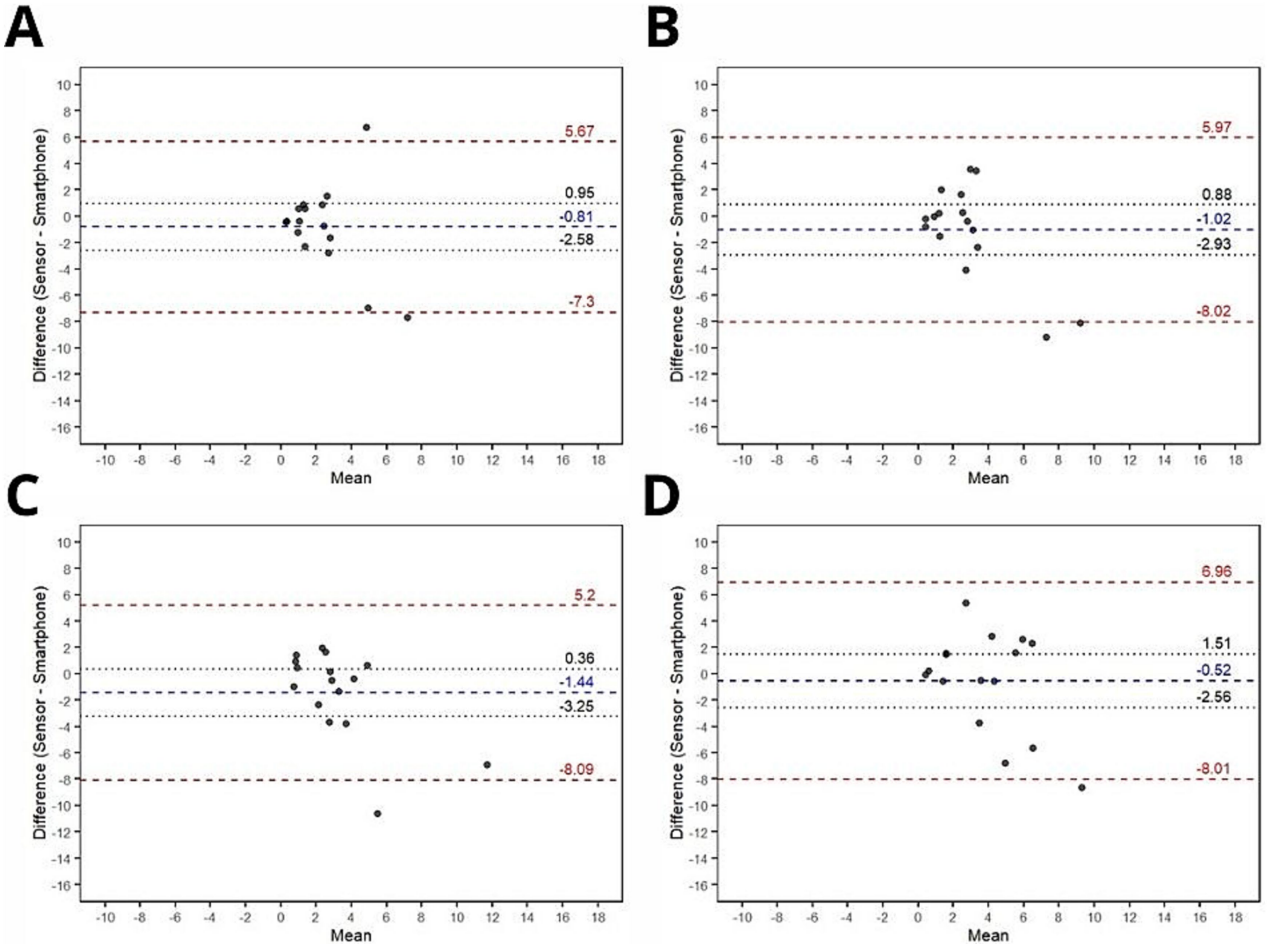
Bland -Altman plots for agreement analysis between joint angle measured from smartphone and ultra-light sensor. **(A)** Relative error (Trial 1); **(B)** Relative error (Trial 2); **(C)** Relative error (Trial 3); **(D)** Relative error (Trial 4). Blue dashed lines: Bias (mean difference). Red dashed lines: Limits of agreement (mean difference ± 1.96 SD). Black dotted lines: 95% confidence interval.

Bland–Altman plots for joint angle, absolute error and relative error are shown in Figures 3–5, respectively.

#### Joint angle

Bland–Altman agreement analysis was conducted on joint angle measurements obtained from both devices across five conditions: baseline, trial #1, trial #2, trial #3, and trial #4. The analysis revealed that the range of agreement was quantitatively narrower under the baseline condition (mean bias ± limits of agreement = 0.23 ± 5.35) compared to conditions involving active movements (trial #1: mean bias mean bias ± limits of agreement = −2.17 ± 8.56; trial #2: mean bias ± limits of agreement = −2.90 ± 7.13; trial #3: mean bias ± limits of agreement = −2.56 ± 8.27; trial #4: mean bias ± limits of agreement = −2.21 ± 9.57). Notably, no significant systematic bias was detected in the baseline, trial #1, or trial #4 conditions. However, trials #2 and #3 exhibited statistically significant biases, with a consistent tendency for the smartphone to yield higher angle values compared to the reference device.

#### Absolute error

Bland–Altman agreement analysis was also performed on absolute error values calculated from both devices across four conditions: trial #1, trial #2, trial #3, and trial #4. The results indicated a consistent range of agreement across all conditions (trial #1: mean bias mean bias ± limits of agreement = −0.81 ± 6.48; trial #2: mean bias ± limits of agreement = −1.02 ± 7; trial #3: mean bias ± limits of agreement = −1.44 ± 6.65; trial #4: mean bias ± limits of agreement = −0.52 ± 7.49). No significant systematic bias was observed in any of the conditions, suggesting a high level of agreement between the devices in terms of absolute error.

#### Relative error

Bland–Altman agreement analysis was also conducted on the relative error values derived from both devices across four conditions: trial #1, trial #2, trial #3, and trial #4. The analysis revealed a consistent range of agreement across condition (trial #1: mean bias mean bias ± limits of agreement = −2.4 ± 7.36; trial #2: mean bias ± limits of agreement = −3.13 ± 5.87; trial #3: mean bias ± limits of agreement = −2.8 ± 7.27; trial #4: mean bias ± limits of agreement = −2.44 ± 8.05). In all conditions, statistically significant systematic biases were observed, consistently favoring the smartphone measurements over the ultra-light sensor.

### Inter-device reliability

The reliability between devices, assessed using ICC (2,1), is presented in Table 2. Joint angle reliability was not significant for the baseline and T1 conditions. However, it was significant for the T2 to T4 conditions, showing moderate reliability at T2 and T3, and good reliability at T4. For absolute error, a significant moderate reliability was observed only under the T3 condition. Regarding relative error, significant moderate reliability was found for both T3 and T4 conditions.

**Table 2:** Intraclass correlation coefficient estimates for measurements between devices.

Conditions	ICC (2,1)	*p*-value
Joint angle		
Baseline	0.432 (−0.728; 0.805)	0.15
T1	0.431 (−0.407; 0.79)	0.11
T2	0.668 (−0.089; 0.891)	0.03*
T3	0.683 (0.077; 0.89)	0.01*
T4	0.725 (0.239; 0.903)	0.006*
Absolute error		
T1	0.238 (−1.152; 0.733)	0.3
T2	0.426 (−0.57; 0.796)	0.13
T3	0.567 (−0.137; 0.844)	0.04*
T4	0.419 (−0.732; 0.8)	0.15
Relative error		
T1	0.381 (−0.44; 0.765)	0.14
T2	0.644 (−0.274; 0.891)	0.06
T3	0.615 (−0.145; 0.869)	0.04*
T4	0.667 (0.052; 0.884)	0.01*

*indicates statistical significance (*p* < 0.05).

### Reliability of the measurements of each device

Table 3 shows the ICC, SEM and MDC values for smartphone and ultra-light sensor. There was significant reliability of the joint angle, absolute error and relative error for both devices. Similar high reliability between devices was found for joint angle, high reliability also was found for absolute error and relative error of the ultra-light wearable sensor and excellent reliability was found for the absolute error and relative error estimated from smartphone.

**Table 3:** Reliability of the joint angle, absolute error and relative error measured by smartphone and ultra-light sensor.

	ICC 3,1 (95% CI)	SEM	MDC	*p*-value
Joint angle				
Smartphone	0.73 (0.55; 0.87)	2.1	5.9	0.001
Wearable	0.7 (0.5; 0.86)	1.8	4.9	0.001
Absolute error				
Smartphone	0.82 (0.67; 0.92)	1.43	3.98	0.001
Wearable	0.44 (0.18; 0.7)	1.15	3.21	0.001
Relative error				
Smartphone	0.78 (0.6; 0.9)	1.78	4.94	0.001
Wearable	0.72 (0.52; 0.87)	1.57	4.37	0.001

## Discussion

It was observed that, in contrast with ultra-light wearable sensor, active joint repositioning performed with smartphones attached to the forearm led participants to adopt more flexed positions compared to the reference position. Additionally, it was noted that the relative errors estimated using smartphones were greater than those estimated using ultra-light wearable sensor in the first trials of active repositioning.

Heroux et al. (2022) suggest that a joint repositioning task, such as the one performed in the present study, represents a high-level judgment, as the joint information from the beginning of the task is sent to higher centers and stored in memory, which must then be retrieved to guide the active joint repositioning. During passive movement, muscle spindles are the primary proprioceptive receptors activated (Matthews, 1972), whereas during active movement, there should be activation of a broader range of proprioceptive sensors, including joint, cutaneous, and muscle receptors (Proske and Weber, 2023).

Authors have shown that knowledge of the initial conditions of joint movement can have significant impacts on performance in joint repositioning tasks (Larish et al., 1984; Inglis et al., 1991). An example of this is the use of vibratory stimulation prior to joint repositioning, which leads to greater errors during the active repositioning task (Larish et al., 1984) when compared to repositioning performed in the absence of vibratory stimulation. In this study, a passive reference position was provided for the participant to memorize, which differs from the proprioceptive conditions established during active joint repositioning while wearing either the ultra-light sensor or the smartphone. Due to its mass, the smartphone is expected to cause greater tissue tensioning and consequent activation of joint, cutaneous, and muscle receptors compared to the ultra-light sensor, altering the initial movement conditions of the reference position, which were stored in memory. The results suggested that the greater the difference between the initial movement information in the reference position and the active repositioning, the greater the active repositioning error.

Our results align with those presented by Roach et al. (2023), in which there was no preference for repositioning the elbow in more flexed or extended positions than the reference position from full extension. In the present study, participants also started from a full extension position to flexion around 90 degrees, and in the smartphone tests, there was a greater tendency to flex the joint beyond the reference position, as seen in the relative error values in the first two attempts. When the test was conducted with ultra-light wearable sensors, the relative errors fluctuated around zero, indicating accurate joint repositioning.

The 90-degree flexion position was primarily chosen because it represents a neutral position for the elbow joint and has minimal gravitational influence. Roach et al. (2023) demonstrated that active repositioning of the elbow from an extended position, such as the one performed in the present study, toward various target angles has little impact on repositioning error values. Among the tested angles, the 90-degree position exhibited the lowest error values, reinforcing its suitability for proprioceptive assessments. Additionally, the 90-degree position was also easier for participants to understand, thereby reducing repositioning errors due to difficulties in comprehending the task.

The replicability of measurements for assessing joint position sense (JPS) during elbow flexion has been investigated using various devices (e.g., Rider and Valdes, 2024: goniometer; Juul-Kristensen et al., 2008: electrogoniometer), with studies employing different flexion angles and reporting moderate-to-good test–retest reliability—with maximum ICC values of 0.59 (Juul-Kristensen et al., 2008) and 0.75 (Rider and Valdes, 2024). Previous research has also employed smartphones to assess JPS during elbow flexion and evaluated measurement replicability (Onoda and Huo, 2016; Ramos et al., 2019). For example, Ramos et al. (2019) assessed the test–retest reliability of JPS evaluation during elbow flexion with a one-week interval between sessions, reporting a highest ICC of 0.69 (moderate reliability) for the 90° flexion condition when considering absolute error values. In the present study, we examined the replicability of joint angle, absolute error, and relative error measurements using both devices. All reliability metrics were statistically significant. ICC values for the smartphone ranged from 0.73 to 0.82, indicating moderate-to-good reliability, while values for the wearable sensor ranged from 0.44 to 0.72, reflecting poor-to-moderate reliability.

Our interpretation of the agreement analysis between devices was based on the mean bias, the width of the limits of agreement, and inter-device reliability. Results showed that the smartphone tended to produce slightly higher values than the wearable, with average differences of no more than 3 units across all evaluated variables, that is a difference that may be considered acceptable. However, although there is no universally accepted standard for limits of agreement in joint position sense (JPS) measurements, a width of up to 5° between devices has been suggested as minimally acceptable for healthy individuals (Oleksy et al., 2022). In our data, this threshold was exceeded. Furthermore, inter-device reliability demonstrated moderate correlations, particularly in the later trials. Considering all these findings, we suggest a cautious interpretation of the agreement between the two devices.

This study may have limitations regarding the number of instrument conditions with different masses. Only two instruments with significantly different mass values were used. Nevertheless, we believe that the chosen smartphone model had a mass very similar to that of many other widely available models on the market. Few models have a mass smaller than 100 grams or much greater than the 173 g of the model used. Additional devices could be added, but we believe this would move beyond real-life conditions. We also do not know how the results could be influenced if other smartphone models and wearables were used. To date, no study has focused on these specific details, and we have no reason to believe that smartphones with masses similar or equal to those we used would generate different results. Another limitation is that this study was conducted with a predominantly young adult population and a small sample size, which prevents the generalization of the results to other populations from different age groups, especially the elderly, who may experience significant changes in proprioception. The study emphasizes the importance of considering the device mass during proprioceptive assessments, highlighting practical implications for the design of portable sensors and smartphones used in clinical settings. Due to their lower mass, lightweight sensors more closely approximate physiological conditions during task performance and may thus have greater potential for application in populations with sensorimotor impairments. In addition to the mass of the devices themselves, fixation elements were required for each device. Specifically, a Velcro strap (~50 g) was necessary for the smartphone, whereas a rubber wristband (11 g) was utilized for the ultra-light sensor. This constitutes a limitation of the present study, particularly because a custom fixation method had to be devised for the smartphone attachment, while the ultra-light sensor could rely on an accessory provided by its manufacturer. We also acknowledge that the use of a fixed order (first the smartphone, followed by the light sensor) may have introduced potential bias, such as learning or fatigue effects, which could have influenced the results, and would be avoided by using a randomized or balanced order. However, the research, despite its limitations, reveals important data that can be addressed in future studies.

The fact that both devices performed measurements that were relatively close or identical reinforces the feasibility of using portable, low-cost technologies for proprioceptive assessment, making this type of evaluation more accessible to a larger portion of the population. The weight of the device appears to be a relevant factor in proprioceptive assessment and interventions. Clinically, the discovery that weight can influence proprioception opens interesting perspectives for more effective interventions, particularly in improving this aspect in individuals who experience reduced proprioception following surgical procedures or pathological conditions. Additional research is recommended to further explore the impact of external factors, such as the device’s weight, on different joints and populations, in order to improve the standardization and reliability of evaluative methods.

## Conclusion

Smartphone and ultra-light sensor had agreements between their measurements and reliable measurements. Inter devices difference in the relative error suggest some influence of the device’s mass on the joint sense position.

## Data Availability

The raw data supporting the conclusions of this article will be made available by the authors without undue reservation.
