# Supplementary material for: Comparison of joint position sense measured by inertial sensors embedded in portable digital devices with different masses
Source: Front Neurosci. 2025 May 13;19:1561241. doi: 10.3389/fnins.2025.1561241 (PMC12106428; doi:10.3389/fnins.2025.1561241)

**Supplementary material**

**Supplementary Figure S1.** Comparison of elbow angle measurements using a smartphone and a 3D motion capture system. (A) Elbow joint angle variation over time measured using a smartphone (red line) and a 3D motion capture system. Measurements were calibrated based on elbow angle during full extension. (B) Bar plot comparing elbow angle measurements from the smartphone and motion capture system, showing a significant difference between the two devices. (C) Representative frames from the video analysis software with anatomical landmarks indicated: wrist (midpoint between radial and ulnar styloid processes), elbow (lateral epicondyle), and shoulder (greater tubercle).

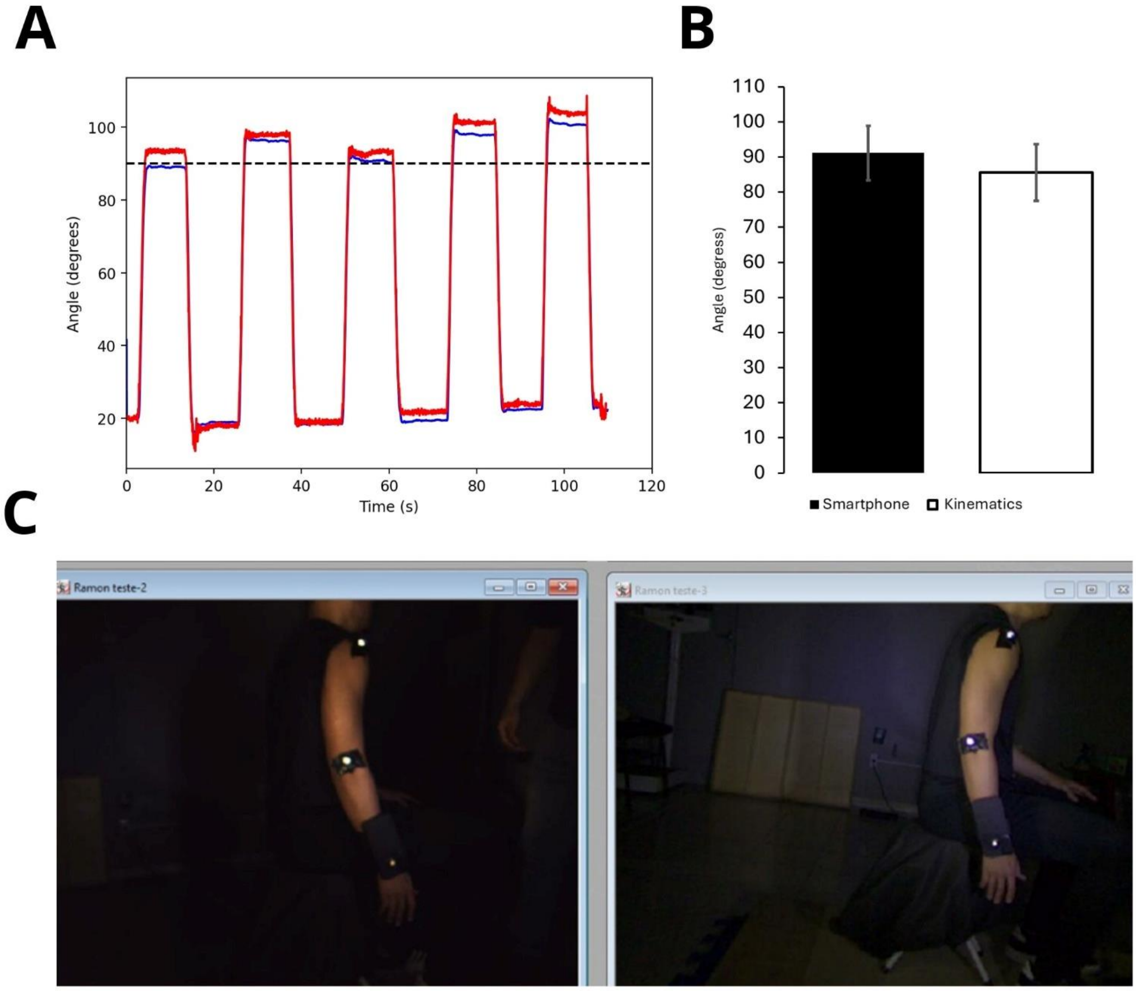

**Supplementary Figure S2.** Joint angle variation measured by inertial sensors from two representative participants (participant 1: panels A and B; participant 2: panels C and D)**. A** and **C** represent the time series, and **B** and **D** represent the spectral distribution of the magnitude across the frequencies. In A and C we plotted the time series filtered using a 40 Hz (black lines), 10 Hz (green lines) and 1 Hz (red lines) low-pass filtering.

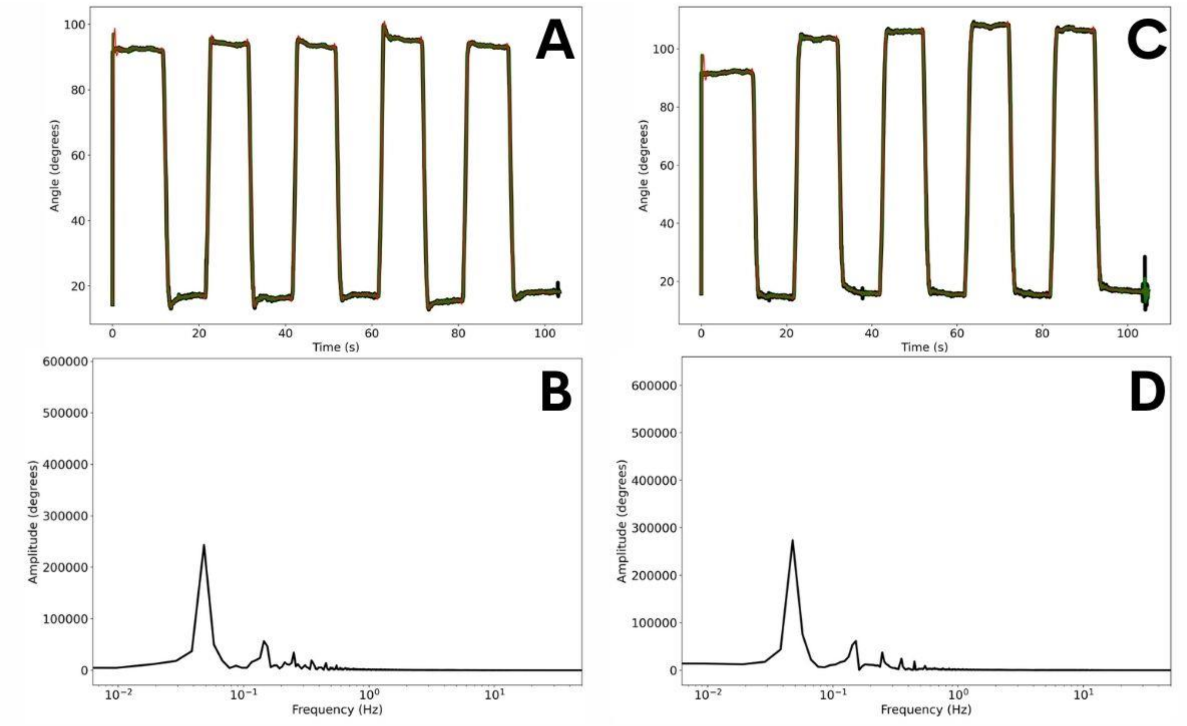

Supplement: Supplementary file 1 [file Data_Sheet_1.pdf]
